# Cervical adenocarcinoma presenting as an ovarian torsion

**DOI:** 10.1016/j.gore.2024.101546

**Published:** 2024-11-19

**Authors:** M. Mvula, S. Roychoudhury, K. King, N. Aravindan, C. Chan, V. John, J. Whyte, GL. Goldberg

**Affiliations:** a Zucker School of Medicine at Hofstra/Northwell, Department of Obstetrics and Gynecology, Northwell Health, New Hyde Park, NY 11040, United States; b Division of Gynecologic Oncology, Department of Obstetrics and Gynecology, Northwell Health, New Hyde Park, NY, United States; c Feinstein Institutes for Medical Research, United States; d Division of Anatomic and Clinical Pathology, Department of Pathology, Northwell Health, New Hyde Park, NY, United States; e Division of Hematology and Oncology, Department of Medicine, Northwell Health, New Hyde Park, NY, United States

**Keywords:** Adenocarcinoma, Cervical cancer, Ovarian torsion, Ovarian cancer

## Abstract

**Background:**

Cervical adenocarcinoma is increasing in incidence and can rarely present as ovarian metastasis.

Case: A 41-year-old female who presented with left lower quadrant pain and vomiting. Intra-operative findings revealed bilateral ovarian cysts with initial pathology results of mucinous ovarian adenocarcinoma. Final histopathology revealed an invasive poorly differentiated mucinous adenocarcinoma of the cervix.

**Conclusions:**

Mucinous adenocarcinoma in the ovary is usually a metastasis and an extensive and comprehensive work-up is required to try to locate the primary tumor.

Précis: Mucinous adenocarcinoma in the ovary is usually a metastasis and an extensive and comprehensive work-up is required to try to locate the primary tumor.

## Highlights

• Cervical adenocarcinoma can present as ovarian metastases that simulate a primary ovarian malignancy, which can lead to misdiagnosis.
• Extensive work-up to locate primary tumor is required in patients with mucinous ovarian adenocarcinoma.
• Cervical adenocarcinoma is increasing in incidence while squamous cell carcinoma is decreasing due to cytologic screening and the HPV vaccine.

## 1 Introduction

Cervical cancer is the fourth most common malignancy affecting women in the world according to the World Health Organization (WHO). In general, cervical neoplasia can be classified into 2 major subtypes: squamous cell carcinoma and adenocarcinoma. Squamous cell carcinoma (SCC) usually arises in the ectocervix while adenocarcinoma (AC) originates from the endocervix. AC comprises roughly 25 % of all cervical cancers. (Hodgson and Park, 2019) While most cervical cancers are of the SCC subtype, the overall incidence of cervical SCC has decreased due to improved liquid cytology screening leading to an increased frequency of AC. Sensitivity of a single liquid cytology screening to detect AC is 45–76 % with a false negative rate of 50 % (Krane et al., 2001).

Most AC (∼80 %) are driven by high-risk human papillomavirus, as noted by both the WHO and the International Endocervical Adenocarcinoma Criteria and Classification (IECC) systems. Recently however, the classification of endocervical AC has shifted to favor that defined by the IECC. The IECC separates cervical AC into two major groups, human papillomavirus-associated (HPVA) and non–HPV-associated (NHPVA), primarily based on morphology. The IECC classification came about to provide a system that was easier to apply in daily practice. It is more reproducible and provides a specific treatment strategy based on the histology and genomic structure as done with other gynecologic cancers.

We present a case of cervical AC presenting as bilateral ovarian masses with ovarian torsion that was initially misdiagnosed as mucinous ovarian adenocarcinoma. While only 6–25 % of ovarian adenocarcinomas are mucinous, it has been reported that 77 % of ovarian mucinous adenocarcinomas are metastatic tumors. (Seidman et al., 2003) The most common primary sites for these mucinous metastases include the gastro-intestinal tract (GI), pancreas or biliary tract, and endocervical AC. In the case of bilateral ovarian tumors, 94 % of these cases are metastatic as opposed to ovarian primary. (Seidman et al., 2003) This case demonstrates ovarian torsion as a unique presentation of cervical adenocarcinoma. This case also highlights the challenges in determining the primary site of mucinous adenocarcinomas in the ovary.

## 2 Case

41-year-old G3P3 female presented to the emergency department (ED) on 9/12/21 with abdominal pain and vomiting. The patient reported left lower quadrant pain, which was described as constant and burning. The pain was not relieved with ibuprofen at home, and she received multiple doses of morphine in the ED to control the pain. She had five episodes of emesis prior to presentation to the ED. She denied any significant medical history and her surgical history included three prior cesarean deliveries. She also denied any other pertinent gynecological history, however, she did report she had not seen a gynecologist since the birth of her last child over 13 years ago.

On physical exam, the patient had left adnexal fullness and left lower quadrant tenderness, without rebound or guarding. Cervix was normal in appearance with no cervical motion tenderness. CT of the abdomen and pelvis revealed bilateral adnexal masses without ascites, peritoneal or omental disease and severe hepatic steatosis. There was a right, primarily solid, adnexal mass, measuring 6 cm in its largest dimension. The larger mass measuring 13.9 cm arose from the left adnexa and was primarily cystic with a lenticular solid component and a daughter cyst anteriorly. Transvaginal ultrasound showed an enlarged right ovary with multiple cysts and large left ovarian cyst with septations and an anterior daughter cyst. The patient was admitted and subsequently taken to the operating room for a diagnostic laparoscopy with a presumptive pre-operative diagnosis of ovarian torsion.

She underwent a laparoscopic bilateral ovarian cystectomy. Intra-operative findings included: a 13 cm left ovarian cyst twisted three times on the left infundibulopelvic ligament with obvious venous congestion. There was a 5 cm right ovarian cyst with intra-operative rupture of clear mucinous fluid and several 1 cm white nodules along the right pelvic side wall near the insertion of the round ligament. Bilateral ovarian cysts, peritoneal washings for cytology were performed at the start of the case, and a right pelvic side wall nodule were sent to pathology. A Pap smear with HPV co-testing was performed prior to the procedure. There were no gross visible lesions on the cervix which was normal size. The pathology results were notable for bilateral mucinous adenocarcinoma, most consistent with ovarian primary. Pelvic side wall nodule pathology showed endosalpingiosis. Atypical pelvic washings, and an elevated CA-19–9 were reported. CA125 and CEA were normal. The Pap smear was negative for intraepithelial malignancy and the HPV was negative.

**Fig. 1:**
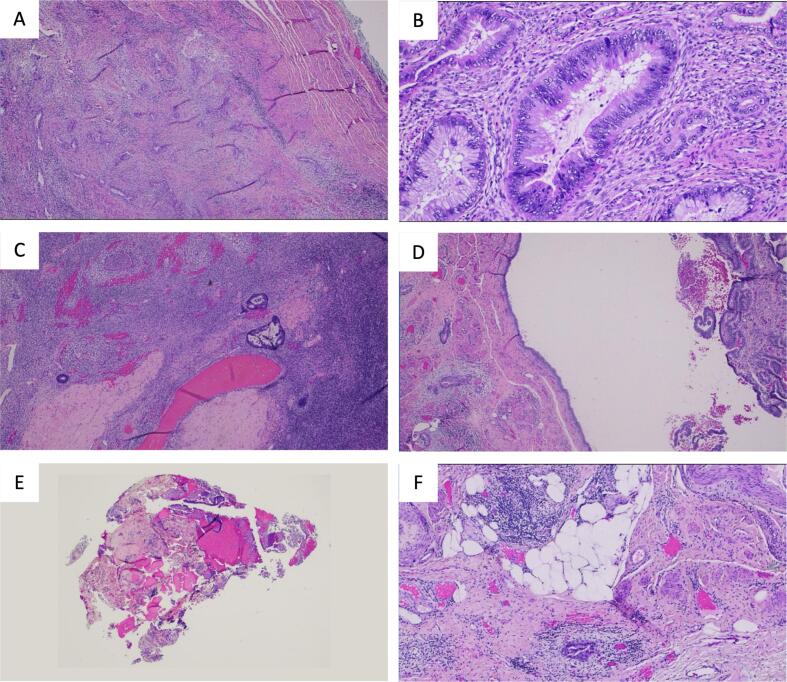
A) Cervical stromal involvement by invasive adenocarcinoma (40X). B) Cervical stromal involvement by invasive adenocarcinoma (200 X) showing high grade nuclear atypia. C) Metastatic adenocarcinoma involving ovary (40x). D) Metastatic adenocarcinoma involving fallopian tube (40x). E) Metastatic adenocarcinoma involving diaphragm (40x). F) Metastatic carcinoma involving parametrium (40x).

The patient was referred to Gynecologic Oncology who noted no cervical lesions, adnexal masses, or uterine/parametrial abnormalities on exam. She was recommended to undergo colonoscopy followed by a staging procedure via a robotic-assisted TLH BSO, omentectomy, pelvic and *para*-aortic lymph node sampling, and an appendectomy. Colonoscopy on 10/21/21 was normal. Intraoperative findings included fine peritoneal nodules throughout the pelvis. The final histopathology from the staging procedure on 11/18/2021 revealed invasive poorly differentiated mucinous adenocarcinoma of the cervix with lymphovascular space involvement, depth of invasion 3.5 cm into cervix extending into the parametrium, and no lymph node involvement, AJCC Stage IVB, PDL1 tumor proportion score (TPS) 1 % (Fig. 1). The patient was subsequently discharged from the hospital, and she underwent 8 cycles of chemotherapy with carboplatin and paclitaxel (1/6/22–––7/15/22, 2/20/23–––4/11/23) as well as immunotherapy with bevacizumab (1/27/22–––7/15/22) and pembrolizumab (2/17/22–––7/15/22). She was kept on maintenance therapy with bevacizumab and pembrolizumab (9/8/22–––10/20/22). She did not receive radiation therapy.

CT on 10/26/22 showed increasing anterior wall and peritoneal implants, consistent with progressive disease. The patient was switched to Tisotumab (12/14/22–2/1/23) and later developed severe ascites requiring hospitalization. While hospitalized, she was switched to carboplatin and taxol. The patient died on 5/13/23, cause of death cardiopulmonary arrest.

## 3 Conclusions

Cervical adenocarcinoma is increasing in incidence and can rarely present as ovarian metastasis. The initial pathology after the first surgical procedure performed was a primary mucinous ovarian adenocarcinoma. Although the frequency of ovarian spread from cervical carcinoma is low, (Bonin et al., 2019) certain patients have increased risk for ovarian metastasis. Shimada et al. showed that the incidence of ovarian metastasis is significantly higher in AC (5.31 %) as compared to SCC (0.79 %). (Shimada et al., 2006) Other risk factors for ovarian metastasis include age greater than 45, FIGO stage, uterine corpus involvement, and unaffected peripheral stromal thickness (<3mm). (Kim et al., 2008) Literature has shown that misdiagnosis can occur, as the histological appearance of ovarian metastases of cervical AC have the tendency to simulate a primary ovarian malignancy, (Reyes et al., 2015) making the differential diagnosis more challenging. There have been other reports of cervical AC with ovarian masses as the primary clinical presentation. Case reports from Liu et al. presented three cases that were similarly misdiagnosed as primary ovarian adenocarcinomas between 1998 and 2016, none of which had primary cervical lesions detected prior to surgery. (Liu et al., 2020) Sun et al. presented a case that also showed advanced endocervical AC that simulated an ovarian primary malignancy. (Sun et al., 2015) Their case included ascites seen on imaging and an intra-operative frozen pathology diagnosis of malignancy at the initial surgery.

In the clinical scenario of bilateral malignant ovarian masses, another primary site cannot be excluded without further workup. Prior studies have demonstrated the ability of a simple algorithm used to pre-operatively distinguish between primary and metastatic ovarian cancers using size and laterality (unilateral ≥ 10 cm implies a primary ovarian neoplasm and a unilateral tumor < 10 cm or bilateral tumors of any size are associated with metastatic tumors. (Yemelyanova et al., 2008) Some authors have explored and proposed more specific and refined algorithms to aid in the primary diagnosis. (Yemelyanova et al., 2008) Additionally, there is no single immunohistochemical marker that can reliably diagnose primary ovarian malignancy, so a panel of multiple markers is suggested. (Dundr et al., 2021) Despite these efforts, the literature remains lacking in evidence for differentiating between a primary and a metastatic mucinous ovarian malignancy in a patient without any clinical evidence of another primary tumor.

The standard of care for AC of the cervix with distant metastases is chemoradiotherapy with targeted therapy. However, there were peculiarities about this case that led to a different course of intervention. In our case, the patient had initial surgery because of an acute clinical presentation consistent with ovarian torsion, which was confirmed intra-operatively and is consistent with current clinical recommendations. Patient then underwent laparoscopic surgery for staging of presumed ovarian cancer with no evidence of extra-ovarian metastases on imaging. While traditionally ovarian cancer is staged via open approach, there is evidence to suggest that patients with stage I ovarian cancer undergoing laparoscopic surgery for staging have shorter operating time, shorter hospital stay, and lower blood loss compared to patients with lararotomy for staging with no significant difference in progression-free survival and overall survival. (Ran et al., 2022) For treatment of advanced cervical cancer, it has been reported by Nagel et al. that surgical removal of adnexal masses can optimize treatment outcomes. (Nagel et al., 2014) Their study found that 23 % of surgically managed patients versus 57 % of expectantly managed patients had disease recurrence. Nevertheless, the treatment strategies for advanced cervical and ovarian cancers are distinct. Although the case presented is rare, it serves as an example of making advancements in our clinical ability to make an accurate pre-operative diagnosis.

Our patient had pathology with PD-L1 with TPS 1 % and was subsequently treated with pembrolizumab. Pembrolizumab is a monoclonal antibody that blocks programmed death 1 (PD-1) receptor on T cells. In a phase 3 trial of patients with cervical cancer, the addition of pembrolizumab reduced disease progression by 35 % and reduced death by 36 % in patients with a PD-L1 positive score > 1. In this study, most patients had squamous cell carcinoma; 18 % of patients in the pembrolizumab group had adenocarcinoma and 27 % percent in the placebo group. (Colombo et al., 2021) PD-L1 positivity is higher in squamous cell carcinoma as compared to adenocarcinoma. (Reddy et al., 2017) While PD-L1 is less common in adenocarcinoma, PD-L1 testing is necessary as TPS is a marker for efficacy of monoclonal antibodies such as pembrolizumab.

In summary, cervical AC is increasing in incidence while SCC is decreasing due to cytologic screening and the HPV vaccine. However, mucinous ovarian AC is usually a metastasis and an extensive and comprehensive work-up is required to try to locate the primary tumor.

Consent: Written informed consent was obtained from the patient for publication of this case report and accompanying images. A copy of the written consent is available for review by the Editor-in-Chief of this journal on request.

## CRediT authorship contribution statement

**M. Mvula:** Writing – original draft. **S. Roychoudhury:** Data curation. **K. King:** Writing – review & editing. **N. Aravindan:** Writing – review & editing. **C. Chan:** Data curation. **V. John:** Data curation. **J. Whyte:** Data curation. **GL. Goldberg:** Supervision, Investigation.

## Declaration of competing interest

The authors declare that they have no known competing financial interests or personal relationships that could have appeared to influence the work reported in this paper.
